# Genome Sequences of Mycobacteriophages AlanGrant, Baee, Corofin, OrangeOswald, and Vincenzo, New Members of Cluster B

**DOI:** 10.1128/genomeA.00586-15

**Published:** 2015-06-18

**Authors:** Welkin H. Pope, Maria E. Carbonara, Hanna M. Cioffi, Tyler Cruz, Brian Q. Dang, Alexander N. Doyle, Olivia H. Fan, Molly Gallagher, Gabrielle M. Gentile, Brian A. German, Margaret E. Farrell, Madeline Gerwig, Kelsey L. Hunter, Virginia E. Lefever, Nicole A. Marfisi, Jill E. McDonnell, Jappmann K. Monga, Kevin G. Quiroz, Alexandra C. Pong, Patrick A. Rimple, Michelle Situ, Peri C. Sohnen, Annmarie N. Stockinger, Paige K. Thompson, Nicole M. Torchio, Chelsea L. Toner, Megan C. Ulbrich, Neelam I. Vohra, Aala Zakir, Nancy L. Adkins, Bryony R. Brown, Bryce M. Churilla, Zachary J. Kramer, Jonathan S. Lapin, Matthew T. Montgomery, Ashley K. Prout, Sarah R. Grubb, Marcie H. Warner, Charles A. Bowman, Daniel A. Russell, Graham F. Hatfull

**Affiliations:** Department of Biological Sciences, University of Pittsburgh, Pittsburgh, Pennsylvania, USA

## Abstract

AlanGrant, Baee, Corofin, OrangeOswald, and Vincenzo are newly isolated phages of *Mycobacterium smegmatis* mc^2^155 discovered in Pittsburgh, Pennsylvania, USA. All five phages share nucleotide similarity with cluster B mycobacteriophages but span considerable diversity with Corofin and OrangeOswald in subcluster B3, AlanGrant and Vincenzo in subcluster B4, and Baee in subcluster B5.

## GENOME ANNOUNCEMENT

Mycobacteriophages can be readily isolated from environmental samples such as soil and compost using *Mycobacterium smegmatis* mc^2^155 as a host (1). Comparative genomics of several hundred individually isolated phages reveals them to be highly diverse, and there are at least 30 types sharing no extensive DNA sequence similarity with each other (2). Most of these phages were isolated and characterized by participants in the Science Education Alliance Phage Hunters Advancing Genomics and Evolutionary Science (SEA-PHAGES) program, which provides freshman students with authentic research experiences (3).

Phages Baee, OrangeOswald, AlanGrant, Corofin, and Vincenzo were isolated using soil samples from the Pittsburgh, Pennsylvania, area, the first two by direct plating on *M. smegmatis* mc^2^155, and the other three following enrichment with the same strain. Following purification, amplification, and DNA extraction, genomes were sequenced on an Illumina MiSeq platform using 140-bp single-end reads. Reads were assembled using Newbler to yield major contigs of 72,109 bp and 68.9% G+C content, with 1,147-fold coverage (AlanGrant); 70,270 bp and 67.6% G+C content, with 1,007-fold coverage (Baee); 68,685 bp and 67.5% G+C content, with 486-fold coverage (Corofin); 68,674 bp, 67.5% G+C content, with 888-fold coverage (OrangeOswald); and 72,189 bp and 68.9% G+C content, with 194-fold coverage (Vincenzo). All genomes are circularly permuted and were bioinformatically linearized to align with other related genomes. Protein-coding genes were predicted using Glimmer and GeneMark, using both heuristic and *M. smegmatis* models; Aragorn and tRNAscan-SE did not predict tRNAs in any of the genomes. The genomes have 96 to 103 predicted protein-coding genes, of which ~30 in each genome have functions predicted by BLASTp and HHpred.

Comparison with other mycobacteriophages using BLASTn showed extensive nucleotide sequence similarity with phages in cluster B. Comparisons of average nucleotide identities and of genome maps (4) showed that Corofin and OrangeOswald are new members of subcluster B3, with 99% sequence identity to each other, as well as to Akoma and Audrey (5); AlanGrant and Vincenzo are new members of subcluster B4, with 99% sequence identity to each other, and 83% identity to Nigel (6), spanning 90% of the genomes. Baee is a member of subcluster B5, with 90% nucleotide identity to Acadian over 96% of their genome lengths.

Approximately 30% of the genes in each genome are homologues (40 to 70% amino acid identity) of *M. abscessus* putative prophage genes (7). These include virion structural genes, endolysins, RNaseE, and many genes of unknown function, and are components of at least five different putative *M. abscessus* prophages. The cluster B phages are not temperate and do not encode integrases or putative repressors, but these relationships suggest that there are closely related temperate phages infecting *M. abscessus* that the cluster B’s have been exchanging genes with in relatively recent evolutionary time. We note that some cluster B phages have narrow host ranges and do not efficiently infect other *M. smegmatis* strains (8), and it is not known if the phages isolated here or any of the cluster B phages infect *M. abscessus*.

### Nucleotide sequence accession numbers.

The AlanGrant, Baee, Corofin, OrangeOswald, and Vincenzo genome sequences are available from GenBank under the accession numbers KR080200, KR080199, KR080205, KR080203, and KR080194, respectively.
